# Effects of the SGLT2 Inhibition on Cardiac Remodeling in Streptozotocin-Induced Diabetic Rats, a Model of Type 1 Diabetes Mellitus

**DOI:** 10.3390/antiox11050982

**Published:** 2022-05-17

**Authors:** Camila Moreno Rosa, Dijon Henrique Salome Campos, David Rafael Abreu Reyes, Felipe Cesar Damatto, Lucas Yamada Kurosaki, Luana Urbano Pagan, Mariana Janini Gomes, Camila Renata Corrêa, Ana Angelica Henrique Fernandes, Marina Politi Okoshi, Katashi Okoshi

**Affiliations:** 1 Department of Internal Medicine, Botucatu Medical School, Sao Paulo State University, UNESP, Botucatu 18618-687, SP, Brazil; camilabasseto@prof.educacao.sp.gov.br (C.M.R.); dijon.campos@unesp.br (D.H.S.C.); david.reyes@unesp.br (D.R.A.R.); felipe.damatto@unesp.br (F.C.D.); lucas.kurosaki@unesp.br (L.Y.K.); luana.pagan@unesp.br (L.U.P.); marina.okoshi@unesp.br (M.P.O.); 2 Brigham and Women’s Hospital, Harvard Medical School, Boston, MA 02115, USA; mgomes18@bwh.harvard.edu; 3 Department of Pathology, Botucatu Medical School, Sao Paulo State University, UNESP, Botucatu 18618-689, SP, Brazil; camila.camacho@unesp.br; 4 Department of Chemistry and Biochemistry, Institute of Biosciences, Sao Paulo State University, UNESP, Botucatu 18618-970, SP, Brazil; ana.ah.fernandes@unesp.br

**Keywords:** SGLT2 inhibitor, ventricular remodeling, oxidative stress, myocardial fibrosis, dapagliflozin, cardiac function

## Abstract

Clinical trials have shown that sodium glucose co-transporter 2 (SGLT2) inhibitors improve clinical outcomes in diabetes mellitus (DM) patients. As most studies were performed in Type 2 DM, the cardiovascular effects of SGLT2 inhibition still require clarification in Type 1 DM. We analyzed the effects of SGLT2 inhibitor dapagliflozin on cardiac remodeling in rats with streptozotocin-induced diabetes, an experimental model of Type 1 DM. Methods: Male Wistar rats were assigned into four groups: control (C, *n* = 14); control treated with dapagliflozin (C + DAPA, *n* = 14); diabetes (DM, *n* = 20); and diabetes treated with dapagliflozin (DM + DAPA, *n* = 20) for 8 weeks. Dapagliflozin dosage was 5 mg/kg/day. Statistical analyses: ANOVA and Tukey or Kruskal–Wallis and Dunn. Results: DM + DAPA presented decreased blood pressure and glycemia and increased body weight compared to DM (C 507 ± 52; C + DAPA 474 ± 50; DM 381 ± 52 *; DM + DAPA 430 ± 48 # g; * *p* < 0.05 vs. C; # *p* < 0.05 vs. C + DAPA and DM + DAPA). DM echocardiogram presented left ventricular and left atrium dilation with impaired systolic and diastolic function. Cardiac changes were attenuated by dapagliflozin. Myocardial hydroxyproline concentration and interstitial collagen fraction did not differ between groups. The expression of Type III collagen was lower in DM and DM + DAPA than their controls. Type I collagen expression and Type I-to-III collagen ratio were lower in DM + DAPA than C + DAPA. DM + DAPA had lower lipid hydroperoxide concentration (C 275 ± 42; C + DAPA 299 ± 50; DM 385 ± 54 *; DM + DAPA 304 ± 40 # nmol/g tissue; * *p* < 0.05 vs. C; # *p* < 0.05 vs. DM) and higher superoxide dismutase and glutathione peroxidase activity than DM. Advanced glycation end products did not differ between groups. Conclusion: Dapagliflozin is safe, increases body weight, decreases glycemia and oxidative stress, and attenuates cardiac remodeling in an experimental rat model of Type 1 diabetes mellitus.

## 1. Introduction

Diabetes mellitus (DM) is a chronic worldwide pandemic [1]. Patients with DM have a 2.5 times higher risk for developing heart failure than non-diabetic individuals [2]. DM may cause heart failure from ischemic cardiac disease and diabetic cardiomyopathy [1,2]. Diabetic cardiomyopathy is characterized by pathological cardiac changes that can result in heart failure in the absence of systemic arterial hypertension, and structural heart diseases such as coronary artery disease or valvular heart disease [3].

Several mechanisms are involved in diabetic cardiomyopathy [4]. Hyperglycemia increases oxidative stress and advanced glycation end products (AGE) which lead to deleterious systemic and cardiac effects [5]. Furthermore, AGE increases oxidative stress making an interplay between oxidative stress and AGE formation [6,7]. Both AGE and increased oxidative stress induce changes in collagen cross-linking molecules collaborating to reduce cardiac elasticity [4].

Currently, there is no specific treatment to prevent or treat diabetic cardiomyopathy. A new class of antidiabetic drug, selective sodium-glucose co-transporter 2 (SGLT2) inhibitors, has been introduced in clinical practice in the last decade [8]. SGLT2 inhibitors reduce renal glucose reabsorption by inhibiting sodium-glucose co-transporter 2, thus decreasing plasma glucose levels [8]. SGLT2, which is predominantly located in the S1 segment of the proximal tubule, is the main glucose transporter in the kidney and is responsible for 90% of reabsorbed glucose [9]. As well as renal effects, clinical trials have shown that SGLT2 inhibition is associated with important cardiovascular benefits, such as a decrease in cardiovascular death and hospitalization for heart failure in both diabetic and non-diabetic patients, and those with and without heart failure [10,11,12,13]. Although different possibilities have been suggested to explain the cardiovascular effects of SGLT2 inhibitors, their protection mechanisms are not completely understood [12].

Most studies have evaluated the effect of SGLT2 inhibitors on Type 2 DM but few have assessed their effects on Type 1 DM [14,15,16,17,18,19]; also, their effects on cardiovascular diseases are still unclear in Type 1 DM in both clinical and experimental scenarios. In Type 1 DM, the main mechanism responsible for cardiomyopathy is related to the decreased insulin signaling [20]. On the other hand, Type 2 DM-induced cardiomyopathy is mostly related to the development of hyperinsulinemia and cardiac insulin resistance [20]. This study analyzed the effects of SGLT2 inhibitor dapagliflozin on cardiac remodeling in rats with streptozotocin-induced diabetes, an experimental model of Type 1 diabetes mellitus. As possible cardiac injury mechanisms, we assessed myocardial collagen content and crosslinking and oxidative stress including AGEs formation.

Most studies have evaluated the effect of SGLT2 inhibitors on Type 2 DM, but few have assessed their effects on Type 1 DM [14,15,16,17,18,19]; also, their effects on cardiovascular diseases are still unclear in Type 1 DM in both clinical and experimental scenarios. In this study we evaluated the effects of SGLT2 inhibitor dapagliflozin on cardiac remodeling in rats with streptozotocin-induced diabetes, an experimental model of Type 1 diabetes mellitus. As possible cardiac injury mechanisms, we assessed myocardial collagen content and crosslinking and oxidative stress including AGEs formation.

## 2. Materials and Methods

### 2.1. Experimental Groups

Male Wistar rats weighing approximately 450 g were purchased from the Central Animal House at Botucatu Medical School, Sao Paulo State University, UNESP. All animals were housed in individual cages in a room under temperature control at 23 °C and kept on a 12-h light/dark cycle. Water and food were supplied ad libitum. All experiments and procedures were approved by the Ethics Committee of Botucatu Medical School, UNESP.

The rats were assigned into four groups: control (C, *n* = 14); control treated with dapagliflozin (C + DAPA, *n* = 14); diabetes (DM, *n* = 20); and diabetes treated with dapagliflozin (DM + DAPA, *n* = 20).

Diabetes was induced by an intraperitoneal injection of streptozotocin (Sigma, St. Louis, MO, USA) at 40 mg/kg body weight diluted in 0.01 M citrate buffer pH 4.5 [21,22]. Control groups were injected intraperitoneally with the same volume of vehicle. A glucometer (Advantage^®^) was used to measure blood glucose seven days after streptozotocin administration. Rats with glycemia higher than 220 mg/dL were included in the study [23,24]. Dapagliflozin (Bristol Myers Squibb Farmaceutica, Portuguesa, SA, Brazil) was added to rat chow at a dosage of 5 mg/kg/day for 8 weeks. To adjust dapagliflozin dose, chow consumption was measured daily and body weight weekly.

Systolic arterial pressure was measured using the tail-cuff method with a 709-0610 electro-sphygmomanometer (Narco Bio-System^®^, International Biomedical Inc., Austin, TX, USA) [25,26] at the end of the experiment. Cardiac hypertrophy was assessed by right ventricle, left ventricle (LV), and atria weights in both absolute and normalized to body weight values [27,28].

### 2.2. Echocardiographic Study

#### 2.2.1. M-Mode

At the end of the experiment, an echocardiogram was performed with an echocardiograph (General Electric Medical Systems, Vivid S6, Tirat Carmel, Israel) using a 5–11.5 MHz multifrequency probe, as described in our laboratory [29,30,31]. Rats were anesthetized by an injection of ketamine (50 mg/kg) and xylazine (0.5 mg/kg) intramuscularly. Images were printed on a thermal printer (Sony UP-890MD) at a speed of 100 mm/s. All cardiac structures were manually measured by the same investigator (KO), who was blinded to the experimental groups. Values were obtained as the mean of at least five cardiac cycles on M-mode tracings. The following structural variables were measured: left atrium (LA) diameter, LV diastolic and systolic dimensions (LVDD and LVSD, respectively), LV diastolic posterior (LVPWT) and septal (LVSWT) wall thickness, and aorta diameter (AO). LV mass (LVM) was calculated using the formula [(LVDD + LVPWT + LVSWT)^3^ – LVDD^3^] × 1.04. LV relative wall thickness (RWT) was calculated by the formula 2 × LVPWT/LVDD. LV systolic function was analyzed by the following parameters: ejection fraction, endocardial fractional shortening (EFS), and posterior wall shortening velocity (PWSV). LV diastolic function was assessed by the variables of early and late diastolic mitral inflow velocities (E and A waves, respectively), E/A ratio, isovolumetric relaxation time (IVRT), and E-wave deceleration time (EDT). A combined evaluation of systolic and diastolic LV function was made using the myocardial performance index (Tei index).

#### 2.2.2. Tissue Doppler Imaging

Tissue Doppler imaging (TDI) was used to assess systolic (S’) and early (E’) and late (A’) diastolic velocity of the mitral annulus, and E/E’ ratio [32].

### 2.3. Histological Analysis

Transverse sections of LV were fixed in formalin and immersed in paraffin. Hematoxylin–eosin-stained sections [33] were used to measure in each LV at least 50 myocyte diameters as the smallest distance between cell borders across the nucleus [34]. Interstitial collagen fraction was evaluated in Sirius red F3BA-stained slides [35] by analyzing 20 microscopic fields [36]. Evaluation was performed in a Leica microscope (magnification 40×) equipped with camera, computer, and an image analysis software (Image-Pro Plus 3.0, Media Cybernetics, Silver Spring, MD, USA).

### 2.4. Myocardial Hydroxyproline Concentration

The concentration of hydroxyproline (HOP) was evaluated in LV to estimate the myocardial collagen content using a colorimetric assay (QuickZyme Hydroxyproline Assay, Leiden, The Netherlands).

### 2.5. Western Blotting

The expression of lysil oxidase (Abcam, LOX1, ab60178) and Type I (Santa Cruz Biotechnology Inc., Santa Cruz, CA, USA, col1a1, sc-8784-r) and Type III collagen (Abcam, Cambridge, UK, col3a1, ab6310) was assessed by Western blot [37]. After protein extraction, the samples were separated on polyacrylamide gel and transferred to a nitrocellulose membrane. Polyacrylamide concentration was 10% for Types I and III collagen, and 12% for lysyl oxidase. After blockade with milk, the membrane was incubated with primary antibodies overnight at 4 °C. Next, the membrane was washed with PBS and Tween 20 and incubated with secondary peroxidase-conjugated antibodies (Santa Cuz Biotechnology, anti-mouse, sc-2005, and anti-rabbit, sc-2004) for 90 min at room temperature. Bound antibodies were detected using ECL Western Blotting Substrate (Pierce Protein Research Products, Rockford, IL, USA). After stripping antibodies from the membrane (Restore Western Blot Stripping Buffer, Pierce Protein Research Products, Rockford, IL, USA), it was incubated with anti-GAPDH antibody (Santa Cruz Biotechnology, GAPDH 6C5, sc-32233). Protein levels were normalized to GAPDH.

### 2.6. Myocardial Oxidative Stress

An LV sample (∼100 mg) was homogenized in phosphate buffer (0.1 M) pH 7.4 and centrifuged at 12,000× *g* for 15 min at 4 °C. The supernatant was used to measure total protein, lipid hydroperoxide, and anti-oxidant enzyme activities as previously described [38,39].

### 2.7. Advanced Glycation End Products

A myocardial sample embedded in paraffin was cut and prepared on slides for immunohistochemical evaluation. Following deparaffinization, antigen was retrieved in citrate buffer (pH 6.0). After the blockade of endogenous peroxidase and proteins, slides were incubated with primary antibody (anti-AGE, ABCAM, ab23722) overnight at 4 °C and secondary antibody (Histofine Simple Stain Rat, *Nichirei,* *414191F*) for 30 min at room temperature. Slides were then stained with 3,3’-diaminobenzidine (DAB) and hematoxylin, dehydrated, and covered with a cover slip. Quantification was performed using a microscope (magnification 40×). Advanced glycation end products were seen as brown and myocytes as lilac in color. Areas containing vessels were excluded from the analysis.

### 2.8. Statistical Analyzes

Results are shown as mean ± standard deviation or median and percentiles in accordance with normal or non-normal distribution, respectively. Variables were compared by analysis of variance (ANOVA) for a 2 × 2 factorial design followed by the Tukey test for normal distribution parameters, or Kruskal–Wallis and Dunn test for non-normal distribution variables. The following comparisons were performed: C + DAPA vs. C; DM vs. C; DM + DAPA vs. DM; and DM + DAPA vs. C + DAPA. For morphological and molecular analyses, samples were chosen randomly. A level *p* < 0.05 was considered statistically significant.

## 3. Results

Table 1 shows body weight, blood pressure, and glycemia values. There were no differences between groups before DM in body weight and glycemia. Blood glucose concentration after DM induction and before dapagliflozin treatment did not differ between DM + DAPA and DM groups. At the end of the study, body weight was lower and blood pressure and glycemia were higher in DM than C. DM + DAPA had greater body weight and lower blood pressure and glycemia than DM.

**Figure 1:**
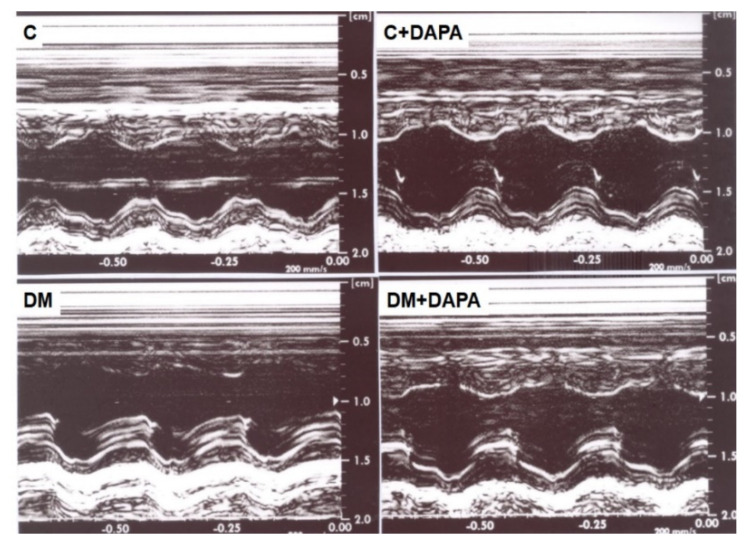
Illustrative left ventricle (LV) M−mode echocardiograms. C: control; C + DAPA: control treated with dapagliflozin; DM: diabetes mellitus; DM + DAPA: DM treated with dapagliflozin.

Figure 1 shows illustrative LV M-mode echocardiograms from all groups. Echocardiographic structural variables are shown in Table 2. LV systolic diameter and LV mass-to-body weight ratio were higher in DM and DM + DAPA than their controls. Left atrium diameter, in absolute or normalized values, and LV diastolic diameter-to-body weight ratio were higher in DM compared to C and lower in DM + DAPA than DM. These data show that the heart was dilated and remodeled by DM; these changes were attenuated in DM + DAPA group. All systolic function indexes were impaired in DM than C (Table 3). DM + DAPA had impaired ejection fraction compared to C + DAPA and a more improved posterior wall shortening velocity and Tei index than DM. In DM + DAPA, endocardial fractional shortening and S’ wave values were between those in C + DAPA and DM and did not differ significantly from either group. LV diastolic function parameters are shown in Table 4. E wave was lower in DM than C. Isovolumic relaxation time in absolute or normalized to heart rate values was higher in DM than C and DM + DAPA.

Anatomical variables are shown in Table 5. LV weight was lower and LV weight-to-body weight ratio higher in DM than C. Right ventricle weight-to-body weight ratio was higher in DM than C and DM-DAPA. The increased right ventricle-to-body weight ratio in DM suggests that the right ventricle was hypertrophied in response to increased LV end-diastolic pressure and pulmonary arterial pressure.

**Figure 2:**
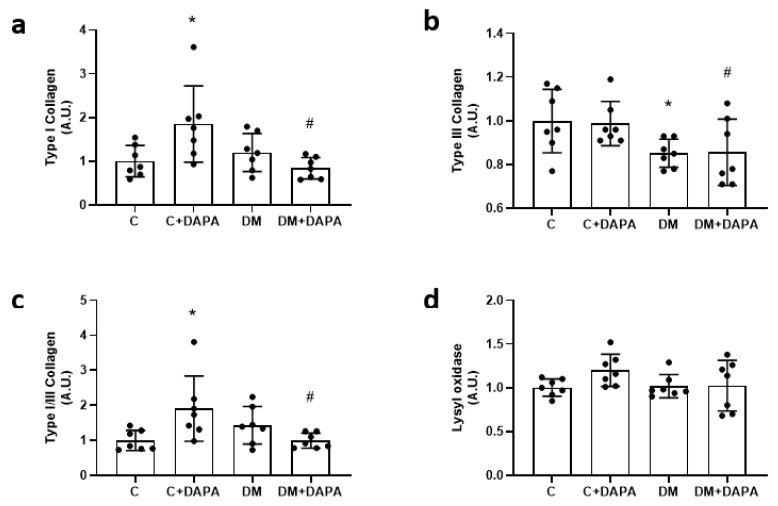
Left ventricular protein expression. Type I collagen (**a**); Type III collagen (**b**); Type I/III collagen ratio (**c**); lysyl oxidase (**d**). Data are mean ± standard deviation. C: control; C + DAPA: control treated with dapagliflozin; DM: diabetes mellitus; DM + DAPA: DM treated with dapagliflozin; ANOVA for a 2 × 2 factorial design and Tukey; * *p* < 0.05 vs. C; ^#^ *p* < 0.05 vs. C + DAPA; sample size is 7 for all groups.

**Figure 3:**
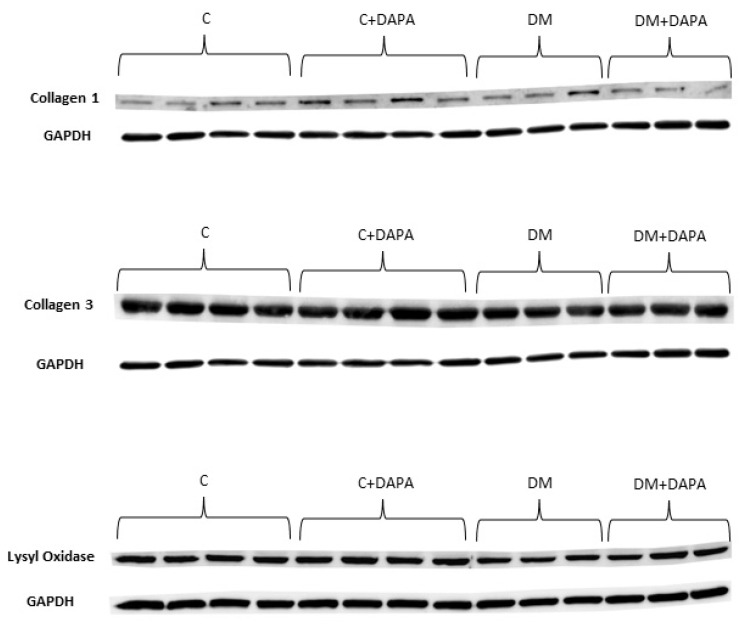
Representative Western blots. C: control; C + DAPA: control treated with dapagliflozin; DM: diabetes mellitus; DM + DAPA: DM treated with dapagliflozin.

Myocardial hydroxyproline concentration and interstitial collagen fraction did not differ between groups. Myocyte diameter was lower in C + DAPA than C (Table 6). Type III collagen expression was lower in DM and DM + DAPA than their controls. Type I collagen expression and Type I-to-type III collagen ratio were higher in C + DAPA than C and DM + DAPA (Figure 2 and Figure 3).

**Figure 4:**
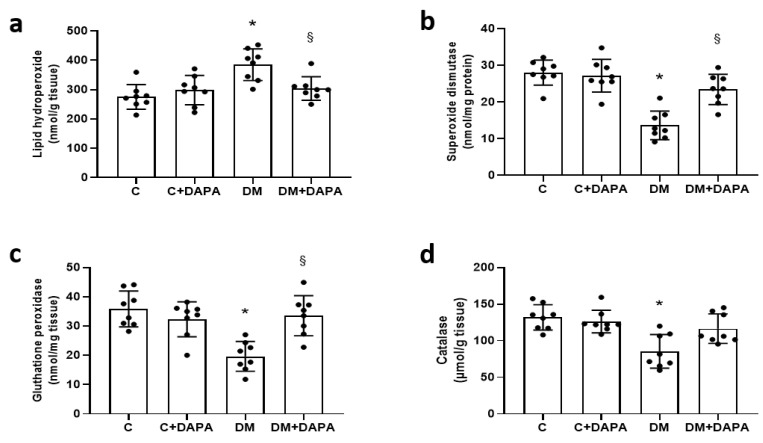
Left ventricular oxidative stress markers. Lipid hydroperoxide (**a**); superoxide dismutase activity (**b**); glutathione peroxidase activity (**c**); and catalase activity (**d**). Data are expressed as mean ± standard deviation. C: control; C + DAPA: control treated with dapagliflozin; DM: diabetes mellitus; DM + DAPA: DM treated with dapagliflozin; ANOVA for a 2 × 2 factorial design and Tukey; * *p* < 0.05 vs. C; ^§^ *p* < 0.05 vs. DM; sample size is 8 for all groups.

DM had higher myocardial lipid hydroperoxide concentration and lower anti-oxidant enzyme activities than C. DM + DAPA had lower lipid hydroperoxide concentration and higher superoxide dismutase and glutathione peroxidase activity than DM (Figure 4). Advanced glycation end products did not differ between groups [C 0.88 (0.67–1.73); C + DAPA 1.29 (0.81–1.66); DM 1.02 (0.71–1.23); DM + DAPA 1.29 (0.80–1.88)%; *p* > 0.05].

## 4. Discussion

Streptozotocin doses between 50 and 80 mg/kg (intravenous or intraperitoneal) have been used to induce Type 1 DM [40,41,42,43,44,45,46]. We have observed in our previous studies [21,22,47] (data not published) that administration of a high streptozotocin dose to rats with great body weights increases glycemia to values higher than 500 mg/dL and leads to a high mortality rate 6–8 weeks after DM induction. In this study, as we have included adult rats with initial body weight around 450 g, we used a streptozotocin dose of 40 mg/kg. Despite the lower dose, glycemia was very high in DM group at the end of the experiment, which shows the success of the model to induce Type 1 DM. In accordance with our results, other authors have succeeded in inducing Type 1 DM in rats with 40 mg/kg streptozotocin [48,49].

As expected, the DM + DAPA group had lower glycemia than DM. SGLT2 inhibitors were first introduced in clinical practice to reduce renal glucose reabsorption and decrease plasma glucose levels. Therefore, clinicians were concerned that loss of urinary glucose could reduce body weight and impair physical status in lean patients with Type 1 DM [14,50]. In fact, only recently has the European Medicines Agency approved SGLT2 inhibition in Type 1 diabetes in addition to the current therapy in individuals with a body mass index ≥ 27 kg/m^2^ [51]. Thus, it was interesting to observe that DM + DAPA had lower glycemia in combination with higher body weight than DM at the end of the experiment. In accordance with our study, diabetic mice treated with SGLT2 inhibitor had increased skeletal muscle mass [52]. The reduced deleterious effects of hyperglycemia may be involved in body mass preservation.

Systolic blood pressure was higher in DM than C and DM + DAPA. Reduced blood pressure has been observed in DM patients after SGLT2 inhibition and attributed to several mechanisms, such as increased natriuresis and decreased plasma volume, arterial stiffness, and sympathetic tone [53,54,55].

In this study, the streptozotocin-induced diabetic cardiomyopathy was well characterized. We have previously showed that this is a good experimental model to assess diabetic cardiomyopathy [21,22,23,24]. DM, despite having lower body weights than C, had increased LV systolic diameter and left atrium diameter. Additionally, left atrium diameter-to-body weight ratio and LV diastolic diameter-to-body weight ratio were approximately 40% greater in DM than C. Both systolic and diastolic function were impaired in the DM group. Dapagliflozin improved LV systolic and diastolic function in the diabetic rats. Improved systolic function has been observed in experimental models of Type 2 DM and other cardiac injury models. For example, dapagliflozin was cardioprotective in angiotensin II-stressed diabetic mice by decreasing fibrosis and inflammation and improving systolic function [56].

Different mechanisms have been proposed to explain the cardioprotective effects of SGLT2 inhibitors, such as an increase in diuresis/natriuresis, autophagy, lysosomal degradation, circulating pro-vascular progenitor cells, erythropoiesis and erythropoietin levels; decrease in blood pressure, oxidative stress, hyperuricemia, inflammation, myocardial fibrosis, and epicardial fat mass; improvement in energy metabolism and vascular function; inhibition of sympathetic nervous system, Na^+^/H^+^-exchanger, and SGLT1; and prevention of ischemia/reperfusion injury [12,56,57]. In this study, we evaluated myocardial collagen content and crosslinking, and oxidative stress including AGEs formation.

Myocardial collagen content evaluated by interstitial collagen fraction and hydroxyproline did not differ between groups, suggesting that myocardial fibrosis is not an important mechanism of cardiac function impairment in this relatively short-term cardiomyopathy model. In accordance with our results, changes in cardiac function were observed before overt myocardial structure alterations in Type 1 diabetes rats [58]. Empagliflozin reduced myocardial fibrosis and cardiac hypertrophy in rats with metabolic syndrome, an experimental model characterized by cardiac hypertrophy and fibrosis [59].

Mechanical myocardial properties are influenced by both the collagen amount and collagen crosslinking. Fibrillar collagen Types I and III are the predominant forms in cardiac extracellular matrix [60]. Type I collagen consists of thick fibers with a high tensile strength, and Type III collagen has small diameter fibers with a low tensile strength [61]. Type 1 collagen was higher in C + DAPA than C. The effects of SGLT2 inhibitors on the normal heart are not completely established. Increased collagen content was observed in healthy mice after ten days with empagliflozin administration [62]. We have not observed differences in Type 1 and Type 3 collagen protein expression between DM + DAPA and DM. Therefore, collagen content or crosslinking was not involved in the differences in ventricular function between DM + DAPA and DM group.

Hyperglycemia increases reactive oxygen species, which are a major mechanism in the pathophysiology of diabetic cardiomyopathy [63,64,65]. DM had higher oxidative stress and reduced anti-oxidant enzyme activity than C and DM + DAPA, showing that dapagliflozin normalized oxidative stress. Other authors have shown normalization of oxidative stress by SGLT2 inhibitors in different models of cardiac injury [56,66]. Arow et al. [56] observed that SGLT2 inhibition-induced decrease in oxygen radicals was accompanied by improved activity of calcium membrane channels with cardioprotective effects similar to those observed in our study. However, there are conflicting results in rodents with streptozotocin-induced DM, with reports of no improvement in antioxidant status after low dose dapagliflozin [67].

Hyperglycemia and oxidative stress increase AGEs. AGEs per se increase oxidative stress and impair ventricular stiffness [6,7,68]. In this study AGEs quantification did not differ between groups. Similarly, empagliflozin did not modulate AGEs production in an ischemia/reperfusion mice model [7]. Our results suggest that increased AGEs formation does not occur after a relatively short period of hyperglycemia. Finally, it should be pointed out that the dapagliflozin-induced reduction in glycemia may have contributed to the decreased oxidative stress and improved cardiac remodeling.

In conclusion, dapagliflozin is safe, increases body weight, decreases glycemia and oxidative stress, and attenuates cardiac remodeling in an experimental model of Type 1 diabetes mellitus. In healthy rats, dapagliflozin increases protein expression of Type 1 collagen.

## Figures and Tables

**Table 1:** Body weight, systolic blood pressure, and blood glucose.

	C (*n* = 14)	C + DAPA (*n* = 14)	DM (*n* = 20)	DM + DAPA (*n* = 20)
Initial BW (g)	448 ± 44	449 ± 44	450 ± 42	448 ± 39
Final BW (g)	507 ± 52	474 ± 50	381 ± 52 *	430 ± 48 ^#§^
Final BP (mmHg)	124 (122–127)	123 (120–126)	140 (136–144) *	133 (130–138) ^#§^
Initial blood glucose (mg/dL)	111 (106–115)	103 (99–111)	111 (104–114)	105 (103–114)
Blood glucose before DAPA	110 (102–113)	108 (104–119)	573 (443–600) *	560 (439–600) ^#^
Final blood glucose (mg/dL)	101 (95–105)	99 (90–109)	494 (422–546) *	145 (131–188) ^#§^

Data are expressed as mean ± standard deviation or median and 25th and 75th percentiles. C: control; C + DAPA: control treated with dapagliflozin; DM: diabetes mellitus; DM + DAPA: DM treated with dapagliflozin; BW: body weight; BP: systolic blood pressure; Blood glucose before DAPA: blood glucose concentration after DM induction and before dapagliflozin treatment. ANOVA for a 2 × 2 factorial design and Tukey or Kruskal–Wallis and Dunn; * *p* < 0.05 vs. C; # *p* < 0.05 vs. C + DAPA; § *p* < 0.05 vs. DM.

**Table 2:** Echocardiographic structural data.

	C (*n* = 14)	C + DAPA (*n* = 14)	DM (*n* = 20)	DM + DAPA (*n* = 20)
HR (bpm)	273 ± 35	251 ± 43	248 ± 33	254 ± 39
LVDD (mm)	7.78 (7.64–7.83)	7.69 (7.46–8.03)	7.88 (7.56–8.37)	8.09 (7.56–8.34)
LVSD (mm)	3.68 ± 0.40	3.88 ± 0.35	4.36 ± 0.53 *	4.30 ± 0.56 ^#^
LVPWT (mm)	1.30 (1.27–1.37)	1.33 (1.30–1.37)	1.33 (1.29–1.39)	1.37 (1.33–1.40)
LVSWT (mm)	1.30 (1.27–1.37)	1.33 (1.30–1.37)	1.35 (1.30–1.39)	1.37 (1.33–1.40)
AO (mm)	4.01 (4.01–4.16)	4.01 (3.83–4.10)	3.83 (3.83–3.89) *	4.01 (3.83–4.01)
LA (mm)	5.62 ± 0.34	5.52 ± 0.44	5.99 ± 0.42 *	5.34 ± 0.44 ^§^
LA/AO	1.39 ± 0.08	1.39 ± 0.06	1.55 ± 0.10 *	1.37 ± 0.09 ^§^
LVDD/BW (mm/kg)	15.4 (15.0–15.8)	15.9 (15.3–18.1)	21.4 (19.7–23.0) *	19.5 (17.0–20.3) ^#§^
LA/BW (mm/kg)	11.2 ± 1.41	11.7 ± 1.18	15.9 ± 1.93 *	12.6 ± 1.36 ^§^
LV mass (g)	0.69 ± 0.07	0.68 ± 0.07	0.72 ± 0.10	0.75 ± 0.09 ^#^
LV mass/BW (g/kg)	1.36 (1.31–1.42)	1.47 (1.30–1.50)	1.95 (1.73–2.11) *	1.79 (1.52–2.03) ^#^
RWT	0.34 ± 0.01	0.35 ± 0.02	0.34 ± 0.02	0.34 ± 0.02

Data are expressed as mean ± standard deviation or median and 25th and 75th percentiles. C: control; C + DAPA: control treated with dapagliflozin; DM: diabetes mellitus; DM + DAPA: DM treated with dapagliflozin; HR: heart rate; LVDD and LVSD: left ventricular (LV) diastolic and systolic diameters, respectively; LVPWT: LV posterior wall thickness; LVSWT: LV septal wall thickness; AO: aorta diameter; LA: left atrial diameter; RWT: relative wall thickness. ANOVA for a 2 × 2 factorial design and Tukey or Kruskal–Wallis and Dunn; * *p* < 0.05 vs. C; ^#^ *p* < 0.05 vs. C + DAPA; ^§^ *p* < 0.05 vs. DM.

**Table 3:** Echocardiographic data for left ventricular systolic function.

	C (*n* = 14)	C + DAPA (*n* = 14)	DM (*n* = 20)	DM + DAPA (*n* = 20)
EFS (%)	52.9 ± 4.51	49.5 ± 3.64	44.9 ± 4.73 *	46.5 ± 4.99
Ejection fraction	0.89 ± 0.03	0.87 ± 0.03	0.83 ± 0.04 *	0.84 ± 0.04 ^#^
PWSV (mm/s)	39.9 ± 4.23	36.3 ± 4.58 *	30.1 ± 3.83 *	33.6 ± 4.85 ^§^
Tei Index	0.48 ± 0.06	0.50 ± 0.06	0.55 ± 0.08 *	0.49 ± 0.07 ^§^
Lateral TDI-S’ (cm/s)	3.50 (3.30–4.00)	3.65 (3.40–4.00)	3.30 (3.10–3.40) *	3.45 (3.10–3.70)
Septal TDI-S’ (cm/s)	3.63 ± 0.50	3.41 ± 0.36	3.22 ± 0.39 *	3.29 ± 0.35
Mean TDI-S’ (cm/s)	3.58 (3.45–3.80)	3.48 (3.35–3.70)	3.20 (3.10–3.40) *	3.35 (3.08–3.68)

Data are expressed as mean ± standard deviation or median and 25th and 75th percentiles. C: control; C + DAPA: control treated with dapagliflozin; DM: diabetes mellitus; DM + DAPA: DM treated with dapagliflozin; EFS: endocardial fractional shortening; PWSV: posterior wall shortening velocity; Tei: myocardial performance index; TDI-S’: tissue Doppler imaging for systolic velocity of the mitral annulus (lateral, septal, and average). ANOVA for a 2 × 2 factorial design and Tukey or Kruskal–Wallis and Dunn; * *p* < 0.05 vs. C; ^#^ *p* < 0.05 vs. C + DAPA; ^§^ *p* < 0.05 vs. DM.

**Table 4:** Echocardiographic data for left ventricular diastolic function.

	C (*n* = 14)	C + DAPA (*n* = 14)	DM (*n* = 20)	DM + DAPA (*n* = 20)
E-wave (cm/s)	76.1 ± 7.75	71.5 ± 3.33	70.5 ± 6.29 *	72.8 ± 6.09
A-wave (cm/s)	46.4 ± 13.7	39.2 ± 6.58	48.5 ± 12.6	44.1 ± 11.9
E/A	1.70 (1.38–1.91)	1.75 (1.67–2.05)	1.48 (1.22–1.74)	1.71 (1.54–2.02)
IVRT (ms)	26.0 ± 2.72	28.5 ± 3.13	38.7 ± 5.67 *	31.1 ± 5.77 ^§^
IVRT/R-R	52.4 (51.4–57.8)	59.4 (57.3–62.3)	80.9 (68.5–87.9) *	61.7 (56.4–66.9) ^§^
EDT (ms)	52.0 ± 8.64	54.5 ± 7.20	54.8 ± 8.57	53.1 ± 7.84
Lateral TDI-E’	4.43 ± 0.65	4.16 ± 0.46	4.30 ± 0.66	4.19 ± 0.62
Septal TDI-E’	4.26 ± 0.69	4.13 ± 0.54	4.22 ± 0.66	4.31 ± 0.78
Average TDI-E’	4.33 (3.85–4.70)	4.10 (3.90–4.40)	4.53 (3.65–4.75)	4.45 (3.78–4.69)
Lateral TDI-A’	3.50 (2.80–4.90)	2.60 (2.30–3.60)	3.85 (3.10–4.30)	3.20 (2.85–3.93)
Septal TDI-A’	3.00 (2.70–4.20)	2.60 (2.10–3.40)	3.50 (2.90–4.60)	2.90 (2.80–4.35)
Average TDI-A’	3.35 (2.80–4.00)	3.20 (2.96–3.99)	3.63 (3.05–4.45)	3.20 (2.96–3.99)
E/average E’	17.7 ± 2.39	17.7 ± 1.57	16.7 ± 2.56	17.2 ± 2.33
Mean E’/average A’	1.29 ± 0.40	1.48 ± 0.42	1.20 ± 0.38	1.30 ± 0.35

Data are expressed as mean ± standard deviation or median and 25th and 75th percentiles. C: control; C + DAPA: control treated with dapagliflozin; DM: diabetes mellitus; DM + DAPA: DM treated with dapagliflozin; E-wave: early diastolic mitral inflow velocity; A-wave: late diastolic mitral inflow velocity; IVRT: isovolumic relaxation time; EDT: E-wave deceleration time; TDI-E’: tissue Doppler imaging (TDI) of mitral annular early velocity (lateral, septal and average between lateral and septal wall velocity); TDI-A’: TDI of mitral annular late velocity (lateral, septal, and average between lateral and septal wall velocity). ANOVA for a 2 × 2 factorial design and Tukey or Kruskal–Wallis and Dunn; * *p* < 0.05 vs. C; ^§^ *p* < 0.05 vs. DM.

**Table 5:** Anatomical data.

	C (*n* = 14)	C + DAPA (*n* = 14)	DM (*n* = 20)	DM + DAPA (*n* = 20)
BW (g)	460 ± 43	449 ± 55	343 ± 52 *	389 ± 46 ^#§^
LV (g)	0.79 ± 0.14	0.79 ± 0.09	0.68 ± 0.11 *	0.72 ± 0.10
LV/BW (g/kg)	1.72 ± 0.32	1.80 ± 0.22	2.01 ± 0.32 *	1.86 ± 0.28
RV (g)	0.24 ± 0.05	0.21 ± 0.03	0.22 ± 0.04	0.20 ± 0.04
RV/BW (g/kg)	0.51 (0.46–0.53)	0.47 (0.41–0.53)	0.62 (0.54–0.67) *	0.52 (0.47–0.57) ^§^
Atria (g)	00.10 ± 0.03	0.08 ± 0.02	0.08 ± 0.02	0.08 ± 0.02
Atria/BW (g/kg)	0.22 ± 0.06	0.19 ± 0.04	0.23 ± 0.06	0.22 ± 0.05

Data are expressed as mean ± standard deviation or median and 25th and 75th percentiles. C: control; C + DAPA: control treated with dapagliflozin; DM: diabetes mellitus; DM + DAPA: DM treated with dapagliflozin; BW: body weight; LV: left ventricle weight; RV: right ventricle weight. ANOVA for a 2 × 2 factorial design and Tukey or Kruskal–Wallis and Dunn; * *p* < 0.05 vs. C; ^#^ *p* < 0.05 vs. C + DAPA; ^§^ *p* < 0.05 vs. DM.

**Table 6:** Myocardial hydroxyproline (HOP) concentration and left ventricular morphometric parameters.

	C (*n* = 10)	C + DAPA (*n* = 10)	DM (*n* = 10)	DM + DAPA (*n* = 10)
HOP (mg/g tissue)	1.43 ± 0.35	1.56 ± 0.54	1.48 ± 0.28	1.50 ± 0.28
ICF (%)	9.41 ± 0.02	8.30 ± 0.02	8.85 ± 0.02	9.88 ± 0.02
Diameter (μm)	17.6 (16.7–18.3)	15.5 (15.1–16.3) *	17.1 (15.6–17.8)	17.6 (15.8–18.1)

Data are expressed as mean ± standard deviation or median and 25th and 75th percentiles. C: control; C + DAPA: control treated with dapagliflozin; DM: diabetes mellitus; DM + DAPA: DM treated with dapagliflozin; ICF: myocardial interstitial collagen fraction; Diameter: myocyte lower diameter. ANOVA for a 2 × 2 factorial design and Tukey or Kruskal–Wallis and Dunn; * *p* < 0.05 vs. C.

## Data Availability

All data generated or analyzed during this study are included in this manuscript.

## Abbreviations

A-wave	late diastolic mitral flow velocity
AGE	advanced glycation end products
ANOVA	analysis of variance
AO	aorta diameter
C	control group
C + DAPA	control group treated with dapagloflozin
DAB	3,3′-diaminobenzidine
DM	diabetes mellitus
DM + DAPA	diabetes mellitus group treated with dapagloflozin
E-wave	early diastolic mitral flow velocity
E/A	ratio
E/E’	ratio
EDT	E-wave deceleration time
EDTA	ethylenediamine tetraacetic acid
EFS	endocardial fraction shortening
GSH-Px	glutathione peroxidase
HOP	hydroxyproline
H_2_O_2_	Hydrogen peroxide
IVRT	isovolumetric relation time
LA	left atrium
LV	left ventricle
LVDD	left ventricle diastolic dimensions
LVPWT	left ventricle posterior wall thickness
LVM	left ventricle mass
LVSWT	left ventricle septal wall thickness
LVSD	left ventricle sistolic dimensions
NADH	nicotinamida adenina dinucleotídeo
PWSV	posterior wall shortening velocity
RWT	left ventricle relative wall thickness
SGLT2	sodium-glucose co-transporter 2
SOD	superoxide dismutase
TDI	tissue Doppler imaging
TDI-A’	tissue Doppler imaging of mitral annular late velocity (lateral, septal and average)
TDI-E’	tissue Doppler imaging of mitral annular early velocity (lateral, septal and average)
TDI-S’	tissue Doppler imaging for systolic velocity of the mitral annulus (lateral, septal and average)
Tei index	myocardial performance index
